# Draft Genome Sequence of Rhodococcus opacus Strain 04-OD7, Which Can Mobilize Phosphate

**DOI:** 10.1128/genomeA.00494-18

**Published:** 2018-06-07

**Authors:** Bang-Xiao Zheng, Huang-Kai Zhang, Kai Ding

**Affiliations:** a Key Laboratory of Urban Environment and Health, Institute of Urban Environment, Chinese Academy of Sciences, Xiamen, People’s Republic of China; b University of Chinese Academy of Sciences, Beijing, People’s Republic of China; c Consejo Superior de Investigaciones Científicas (CSIC), Global Ecology Unit, Centre for Ecological Research and Forestry Applications (CREAF)–CSIC–Universitat Autonoma de Barcelona (UAB), Barcelona, Catalonia, Spain; d CREAF, Cerdanyola del Vallès, Barcelona, Catalonia, Spain; e Xiamen Anjie Medical Data Technology Co. Ltd., Xiamen, People’s Republic of China

## Abstract

Rhodococcus opacus strain 04-OD7 (=CCTCC AB 2017148) is a Gram-positive bacterium showing inorganic phosphate solubilization capacity for the first time in the genus Rhodococcus. We present here the draft genome description of R. opacus 04-OD7 along with multiple phosphorus (P) mobilization-related genes, supporting its inorganic phosphate solubilization.

## GENOME ANNOUNCEMENT

The actinomycete genus Rhodococcus belongs to the family Nocardiaceae, and its number of species has increased dramatically in the last decade (67 species and 57 type strains at the time of this writing, https://www.ezbiocloud.net/, February 2018). R. opacus was commonly seen with lipophilic accumulation ability (1, 2) and even with benzene tolerance (3); however, the phosphate (P) mobilization ability has not been reported before. R. opacus strain 04-OD7 was previously isolated from a fertilized soil sample from an agricultural field in Hailun, China (47°26ʹN, 126°38ʹE), and its calcium phosphate solubilization capacity reached 39.18 ± 6.08 μg liter^−1^ after 96 h of cultivation in Pikovskaya (PVK) medium (4) at 30°C with several organic acid exudations. This strain is a Gram-positive, rod-shaped, and pink-pigmented bacterium. In this study, the draft genome of R. opacus strain 04-OD was characterized.

R. opacus strain 04-OD7 was grown in PVK liquid medium, and the genomic DNA was extracted using a genomic DNA extraction kit for bacteria (BioTeke, Beijing, China) according to the manufacturer’s instructions. Then, the genome was pair end sequenced using the Illumina HiSeq 2000 platform (Novogene, Tianjin, China). *De novo* assembly was performed by SOAP*denovo* version 2.01 (5), obtaining 217 contigs with an *N*_50_ contig length of 112,778 bp. Genome annotations were carried out using the NCBI Prokaryotic Genome Annotation Pipeline (6). The predicted coding DNA sequences (CDSs) were translated into amino acid sequences and submitted to the BLAST, GenBank, Swiss-Prot, Kyoto Encyclopedia of Genes and Genomes (KEGG), Cluster of Orthologous Groups of proteins (COG), and Gene Ontology (G) databases for further analysis.

The draft genome of R. opacus strain 04-OD7 comprises 9,322,710 nucleotides with an average G+C content of 64.47%. A total of 8,884 genes were predicted, of which, 8,407 (94.63%) protein-coding genes, 107 RNA genes (1.20%), and 6,484 functional genes (72.99%) were assigned. Based on COG database alignment, 633, 460, and 875 genes were assigned with amino acid, carbohydrate, and lipid transport and metabolism, respectively. The genome of R. opacus strain 04-OD7 contains genes for exopolyphosphatase (*ppx*), polyphosphate kinase (*ppk*), alkaline phosphatase synthesis response regulator (*pho*P), alkaline phosphatase D (*pho*D), and pyrroloquinoline-quinone synthase (PQQ)-dependent glucose dehydrogenase (*pqq*-*gdh*, GenBank accession number PQP26070), which were all included in the P mobilization and cycles in the environment. By prediction using the alignment with the KEGG database, the coenzyme *pqqC* was also found. The secretion of gluconic acid and 2-keto-d-gluconic acid was considered an effective microbial pathway for enabling inorganic P solubilization (7). The genetic evidence shed light on the involvement of *pqq* operon genes (8, 9). The mutation of any one *pqq* gene would greatly reduce the P solubilizing capacity (10). Hence, *pqq*-*gdh* and *pqq*C would be promising candidates for inorganic P solubilization.

### Accession number(s).

The whole-genome shotgun project has been deposited at DDBJ/ENA/GenBank under the accession number PUIO00000000. The version described in this paper is version PUIO01000000. The raw sequences were deposited in the Sequence Read Archive (SRA) under the accession number SRR6785074.
